# Peri‐operative outcomes of open, laparoscopic and robotic simple prostatectomy

**DOI:** 10.1111/bju.16928

**Published:** 2025-09-16

**Authors:** Nikolaos Pyrgidis, Philipp Weinhold, Gerald Bastian Schulz, Michael Atzler, Leo Federico Stadelmeier, Iason Papadopoulos, Christian Stief, Julian Marcon, Patrick Keller

**Affiliations:** ^1^ Department of Urology University Hospital, LMU Munich Munich Germany

**Keywords:** benign prostatic hyperplasia, minimal‐invasive surgery, prostate enucleation, perioperative outcomes, simple prostatectomy, perioperative outcomes

## Abstract

**Objective:**

To compare peri‐operative outcomes and trends in open (OSP), laparoscopic (LSP) and robot‐assisted simple prostatectomy (RASP).

**Materials and Methods:**

We used German Nationwide Inpatient Data (GRAND), provided by the Research Data Centre of the Federal Bureau of Statistics, and performed multiple patient‐level analyses.

**Results:**

Between 2013 and 2023, 46 234 simple prostatectomies were performed in Germany for benign prostatic hyperplasia without concomitant bladder stones. Of these, 44 194 (96%) were performed with an open, 724 (1%) with a laparoscopic, and 1335 (3%) with a robotic approach. Among patients undergoing OSP, 11 755 (27%) cases were performed with a transcapsular and 32 439 (73%) with a transvesical approach. We compared transcapsular OSP vs RASP and LSP. The adoption of RASP increased exponentially during the period studied, while the use of OSP gradually declined, and that of LSP remained stable. In multivariable regression analyses, in‐hospital transfusions were lower for LSP (6.5%; odds ratio [OR] 0.46, 95% confidence interval [CI] 0.34–0.62, *P* < 0.001) and RASP (7.3%; OR 0.58, 95% CI 0.47–0.72, *P* < 0.001) compared to OSP (13%). In‐hospital urinary retention was significantly less frequent after LSP (4.6%; OR 0.46, 95% CI 0.32–0.65, *P* < 0.001) and RASP (6.4%; OR 0.68, 95% CI 0.54–0.85, *P* < 0.001) compared to OSP (9.3%). The median (interquartile range) hospital stay was 9 (8–12) days for OSP, 7 (6–9) days for RASP (*P* < 0.001) and 4 (4–7) days for LSP (*P* < 0.001). Transcapsular OSP was associated with a lower risk of intensive care unit admission (*P* < 0.001) and shorter hospital stay (*P* < 0.001) compared to transvesical OSP.

**Conclusion:**

Our results showed that RASP is rapidly growing and offers better peri‐operative outcomes compared to OSP.

**Abbreviations**
DRG: Diagnosis Related Groups
ICD‐10‐GM: International Classification of Diseases, 10th Revision, German Modification
IQR: interquartile range
LSP: laparoscopic simple prostatectomy
OPS: German Procedure Classification
OR: odds ratio
OSP: open simple prostatectomy
RASP: robot‐assisted simple prostatectomy

## Introduction

Benign prostatic hyperplasia (BPH) is a common condition affecting aging men, often leading to LUTS that can significantly impair quality of life [1]. For patients who fail to respond or are intolerant to medical therapy, surgical intervention remains the cornerstone of treatment [2]. Traditionally, open simple prostatectomy (OSP) has been the standard procedure for managing larger prostate glands [3]. Nevertheless, accumulating evidence suggests that endoscopic enucleation of the prostate offers better short‐ and long‐term outcomes [4].

With advancements in surgical technology and techniques, minimally invasive approaches such as laparoscopic simple prostatectomy (LSP) and robot‐assisted simple prostatectomy (RASP) have also emerged as viable alternatives, offering similar efficacy and safety compared to endoscopic enucleation of the prostate [5, 6]. However, despite the widespread adoption of robotic surgery in countries such as Germany [7], the cost‐effectiveness and overall benefit of LSP and RASP for both patients and healthcare systems remain areas of debate [8]. The choice of surgical approach is highly healthcare system‐dependent, as financing models and reimbursement structures vary across countries [9]. For example, in single‐payer systems such as the NHS, the widespread use of robotic platforms may be limited unless there is clear evidence of superior outcomes [10]. In contrast, in the United States, the adoption of robotic surgery has been more aggressive [11]. Overall, LSP and RASP are often associated with higher procedural costs, longer operating times, and the need for specialised equipment and training [12]. Furthermore, the choice among open, laparoscopic and robotic approaches often depends on institutional resources, surgeon expertise, and patient preference [13]. Indeed, OSP is still widely implemented and can be performed either through a transvesical (Freyer technique) or a transcapsular (Millin technique) approach [14].

To date, few studies have examined the surgical trends in simple prostatectomy for BPH, or performed direct comparisons of peri‐operative outcomes across these modalities [15]. In this context, we aimed to provide a comprehensive analysis of OSP, LSP and RASP, focusing on peri‐operative outcomes and trends in the surgical management of BPH.

## Methods

### German Nationwide Inpatient Data Registry

We utilised data from the German Nationwide Inpatient Data (GRAND) registry maintained by the Federal Bureau of Statistics in Wiesbaden, Germany. This comprehensive dataset encompasses information on all hospitalised patients in Germany from 2013 to 2023, excluding cases related to military, psychiatric and forensic care [16]. Access to the anonymised dataset was granted following the necessary approvals (LMU – 4710‐2022), and all data were stored at the Research Data Centre of the Federal Bureau of Statistics. Our team worked exclusively with aggregated summary data provided by the Research Data Centre and did not have access to individual patient records. According to German regulations, ethical approval and patient consent were not required for this study.

Since the introduction of the Diagnosis Related Groups (DRG) payment system in 2005, German hospitals have been required to submit patient data on hospital diagnoses, peri‐operative outcomes, and surgical procedures to the Institute for the Hospital Remuneration System. Diagnoses and outcomes are coded using the International Classification of Diseases, 10th Revision, German Modification (ICD‐10‐GM), while surgical procedures are coded using the German Procedure Classification (OPS). The German Institute for Medical Documentation and Information ensures standardised coding practices across the country.

### Data Source

This study included male patients diagnosed with BPH (ICD‐10‐GM: N40) who underwent OSP with the Millin (OPS code: 5‐603.10) or the Freyer technique (OPS code: 5‐603.00), LSP (OPS code: 5‐603.11) or RASP (OPS code: 5‐987) between 2013 and 2023. Patients with concomitant bladder stones were excluded (ICD‐10‐GM: N21.0). Additional ICD‐10‐GM and OPS codes were utilised to collect data on comorbidities and inpatient complications, as previously published [17].

The primary objective of this study was to compare in‐hospital peri‐operative morbidity, including sepsis, postoperative incontinence, urinary retention, transfusion rates, acute kidney injury, and intensive care unit admissions of RASP and LSP vs transcapsular (Millin) OSP for BPH. Secondary analyses examined trends in the use of simple prostatectomy over time, the length of hospital stay after RASP and LSP vs transcapsular (Millin) OSP, as well as the in‐hospital peri‐operative outcomes of transcapsular (Millin) vs transvesical (Freyer) OSP. Urinary retention was defined as the reinsertion of a bladder catheter after its removal during hospitalisation, and urinary incontinence as any involuntary urine loss following catheter removal during hospitalisation. Both outcomes were assessed by the treating clinicians.

### Statistical Analysis

Multivariable logistic and linear regression models were applied to evaluate in‐hospital outcomes. These models were adjusted for key patient characteristics, including age, diabetes, chronic kidney disease, hypertension, and obesity. Categorical variables are reported as frequencies and proportions, while continuous variables are summarised as medians with interquartile ranges (IQRs). Results are presented as odds ratios (ORs) with 95% CIs, and statistical significance was determined at a *P* value threshold of <0.05. Statistical analyses were conducted using scripts developed by our research team and executed by the Research Data Centre (source: Research Data Centre, DRG Statistics 2013–2023). All analyses were performed using R software (version 3.6.3).

## Results

### Baseline Characteristics

**Fig. 1:**
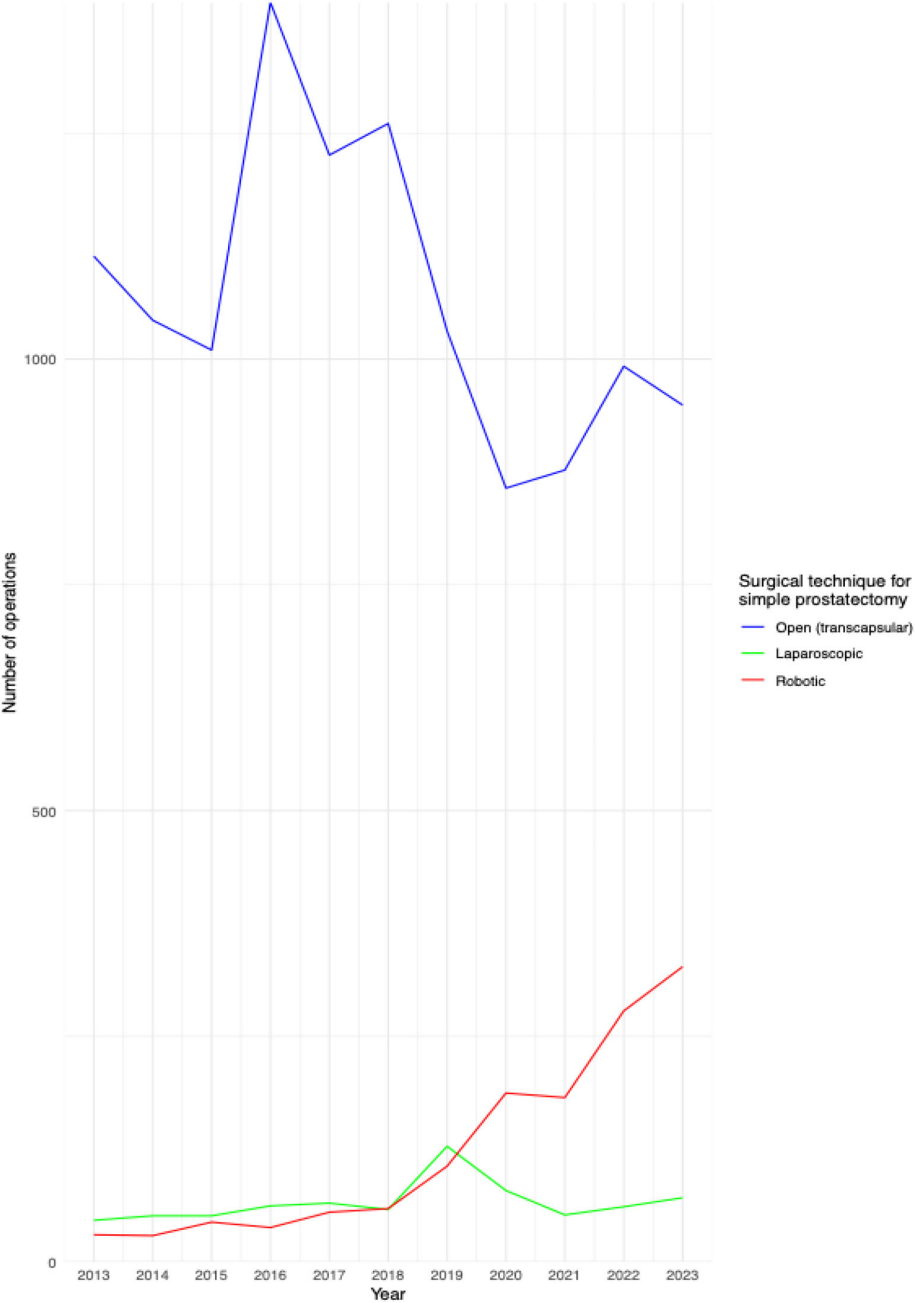
The annual trends of simple prostatectomy based on the surgical approach. All simple prostatectomies were performed via a transcapsular approach.

Between 2013 and 2023, a total of 46 234 simple prostatectomies were performed in Germany for patients with BPH and no concomitant bladder stones. Of these, 44 194 (96%) were performed with an open, 724 (1%) with a laparoscopic, and 1335 (3%) with a robotic approach. Among patients undergoing OSP, 11 755 (27%) cases were performed using a transcapsular approach and 32 439 (73%) using a transvesical approach. Minimally invasive simple prostatectomy was performed only with a transcapsular approach. Therefore, patients undergoing LSP and RASP were compared only with patients undergoing transcapsular OSP. The baseline characteristics of patients undergoing transcapsular OSP, LSP and RASP are summarised in Table 1. In more recent years, the number of RASP procedures increased significantly, while OSP procedures gradually decreased, and LSP remained relatively stable. The annual trends for all surgical approaches for transcapsular simple prostatectomy are illustrated in Fig. 1.

**Table 1:** Baseline characteristics of the included patients.

Characteristic	Overall, *n* = 13 814	Transcapsular OSP, *n* = 11 755	LSP, *n* = 724	RASP, *n* = 1335
Age, years	73 (67–78)	73 (67–78)	74 (67–79)	72 (66–77)
Diabetes, *n* (%)	2552 (18)	2193 (19)	132 (18)	227 (17)
Chronic heart failure, *n* (%)	536 (3.9)	463 (3.9)	38 (5.2)	35 (2.6)
COPD, *n* (%)	620 (4.5)	547 (4.7)	21 (2.9)	52 (3.9)
Chronic kidney disease, *n* (%)	1059 (7.7)	915 (7.8)	61 (8.4)	83 (6.2)
Hypertension, *n* (%)	7796 (56)	6674 (57)	422 (58)	700 (52)
Obesity, *n* (%)	1021 (7.4)	905 (7.7)	42 (5.8)	74 (5.5)
Prostate cancer, *n* (%)	1202 (8.7)	996 (8.5)	66 (9.1)	140 (10)
Year of surgery, *n* (%)				
2013	1190 (8.6)	1114 (9.5)	46 (6.4)	30 (2.2)
2014	1123 (8.1)	1043 (8.9)	51 (7.0)	29 (2.2)
2015	1105 (8.0)	1010 (8.6)	51 (7.0)	44 (3.3)
2016	1495 (11)	1395 (12)	62 (8.6)	38 (2.8)
2017	1346 (9.7)	1226 (10)	65 (9.0)	55 (4.1)
2018	1378 (10.0)	1261 (11)	58 (8.0)	59 (4.4)
2019	1265 (9.2)	1031 (8.8)	128 (18)	106 (7.9)
2020	1123 (8.1)	857 (7.3)	79 (11)	187 (14)
2021	1111 (8.0)	877 (7.5)	52 (7.2)	182 (14)
2022	1331 (9.6)	992 (8.4)	61 (8.4)	278 (21)
2023	1347 (9.8)	949 (8.1)	71 (9.8)	327 (24)

Variables are presented as median (interquartile range) or frequencies with proportions.

COPD, chronic obstructive pulmonary disease; LSP, laparoscopic simple prostatectomy; OSP, open simple prostatectomy; RASP, robot‐assisted simple prostatectomy.

### Peri‐Operative Outcomes of Transcapsular OSP, LSP and RASP

Compared to OSP, LSP and RASP demonstrated significantly better outcomes in terms of in‐hospital transfusion rates based on multivariable analysis. Transfusions were required in 1472 OSP cases (13%), but occurred less frequently in LSP (6.5%; OR 0.46, 95% CI 0.34–0.62, *P* < 0.001) and RASP (7.3%; OR 0.58, 95% CI 0.47–0.72, *P* < 0.001). Compared to OSP, RASP and LSP also demonstrated better outcomes regarding in‐hospital urinary retention. Urinary retention occurred in 1089 OSP patients (9.3%), while rates were significantly lower in LSP (4.6%; 0.46, 95% CI 0.32–0.65, *P* < 0.001) and RASP (6.4%; OR 0.68, 95% CI 0.54–0.85, *P* < 0.001). In particular, RASP, compared to OSP, reduced transfusions by 72% and urinary retentions by 47%. Accordingly, LSP, compared to OSP, reduced transfusions by 117% and urinary retentions by 127%.

Patients undergoing LSP and RASP were discharged earlier compared to OSP. The median (IQR) length of hospital stay was 9 (8–12) days for OSP, 7 (6–9) days for RASP, and 4 (4–7) days for LSP. Both RASP and LSP were associated with a shorter length of hospital stay compared to OSP (RASP: day difference −2.3, 95% CI −2.7 to −1.9, *P* < 0.001; LSP: day difference −4, 95% CI −4.5 to −3.5, *P* < 0.001). By contrast, the incidence of intensive care unit admissions (*P* = 0.8 and *P* = 0.3) in‐hospital incontinence (*P* = 0.2 and *P* = 0.2), acute kidney injury (*P* = 0.6 and *P* = 0.7) and sepsis (*P* = 0.9 and *P* = 0.3) did not differ significantly among OSP, LSP and RASP. All peri‐operative outcomes for transcapsular simple prostatectomy are detailed in Table 2.

**Table 2:** Multivariable linear and logistic regression analysis based on the surgical approach (open vs laparoscopic vs robot‐assisted) for transcapsular simple prostatectomy.

Outcome	Transcapsular OSP		LSP			RASP		
	Cases, *n* (%)	Estimate	Cases, *n* (%)	Estimate (95% CI)	*P*	Cases, *n* (%)	Estimate (95% CI)	*P*
ICU admission	606 (5.2)	–	39 (5.4)	1.04 (0.74 to 1.44)	0.8	58 (4.3)	0.87 (0.65 to 1.13)	0.3
Transfusion	1472 (13)	–	47 (6.5)	**0.46 (0.34 to 0.62)**	**<0.001**	98 (7.3)	**0.58 (0.47 to 0.72)**	**<0.001**
Sepsis	117 (1.0)	–	7 (1.0)	0.96 (0.4 to 1.9)	0.9	9 (0.7)	0.7 (0.33 to 1.3)	0.3
Acute kidney injury	400 (3.4)	–	23 (3.2)	0.9 (0.57 to 1.35)	0.6	39 (2.9)	0.94 (0.66 to 1.3)	0.7
Postoperative incontinence	517 (4.4)	–	24 (3.3)	0.74 (0.47 to 1.1)	0.2	47 (3.5)	0.83 (0.6 to 1.12)	0.2
Urinary retention	1089 (9.3)	–	33 (4.6)	**0.46 (0.32 to 0.65)**	**<0.001**	85 (6.4)	**0.68 (0.54 to 0.85)**	**<0.001**
Length of hospital stay	9 (8–12)	–	4 (4–7)	**‐4 (−4.5 to − 3.5)**	**<0.001**	7 (6–9)	**−2.3 (−2.7 to −1.9)**	**<0.001**

All models are adjusted for age, diabetes, chronic renal failure, hypertension, and obesity. Length of hospital stay is presented as median with interquartile range. The bold cells indicate statistically significant *P* values.

ICU, intensive care unit; LSP, laparoscopic simple prostatectomy; OSP, open simple prostatectomy; RASP, robot‐assisted simple prostatectomy.

### Peri‐Operative Outcomes of Transvesical vs Transcapsular OSP

In the multivariable regression analysis, transvesical and transcapsular OSP showed no significant differences in terms of in‐hospital transfusion rates (13% vs 12%; OR 0.95, 95% CI 0.89–1.01, *P* = 0.09), sepsis rates (1% vs 1%; OR 0.95, 95% CI 0.77–1.16, *P* = 0.6), urinary retention (8.7% vs 8.7%; OR 1.01, 95% CI 0.94–1.09, *P* = 0.7), or postoperative incontinence (4.4% vs 4.3%; OR 0.98, 95% CI 0.88–1.08, *P* = 0.6). However, transcapsular OSP was associated with lower rates of intensive care unit admissions (5.1% vs 6%; OR 0.84, 95% CI 0.77–0.92, *P* < 0.001) compared to transvesical OSP. Additionally, patients undergoing transcapsular OSP stayed in hospital for 0.87 fewer days (9 vs 11 days; 95% CI −0.99 to −0.74, *P* < 0.001) compared to those undergoing transvesical OSP. All peri‐operative outcomes of transvesical vs transcapsular OSP are shown in Table 3.

**Table 3:** Multivariable linear and logistic regression analysis based on the surgical approach (transcapsular vs transvesical) for open simple prostatectomy.

Outcome	Transvesical OSP		Transcapsular OSP		
	Cases, *n* (%)	Estimate	Cases, *n* (%)	Estimate (95% CI)	*P*
ICU admission	1958 (6.0)	–	698 (5.1)	**0.84 (0.77 to 0.92)**	**<0.001**
Transfusion	4062 (13)	–	1609 (12)	0.95 (0.89 to 1.01)	0.09
Sepsis	338 (1.0)	–	132 (1.0)	0.95 (0.77 to 1.16)	0.6
Acute kidney injury	1199 (3.7)	–	458 (3.3)	0.93 (0.83 to 1.04)	0.2
Postoperative incontinence	1439 (4.4)	–	587 (4.3)	0.98 (0.88 to 1.08)	0.6
Urinary retention	2823 (8.7)	–	1203 (8.7)	1.01 (0.94 to 1.09)	0.7
Length of hospital stay	10 (8–13)	–	9.0 (7–11)	**−0.87 (−0.99 to −0.74)**	**<0.001**

All models are adjusted for age, diabetes, chronic renal failure, hypertension, and obesity. Length of hospital stay is presented as median with interquartile range. The bold cells indicate statistically significant *P* values.

ICU, intensive care unit; OSP, open simple prostatectomy.

## Discussion

This nationwide analysis provides valuable insights into the peri‐operative outcomes of OSP, LSP and RASP in Germany. Our findings indicate that RASP and LSP present favourable in‐hospital outcomes compared to OSP. Specifically, both minimally invasive approaches were associated with lower rates of in‐hospital transfusions and urinary retentions, as well as shorter hospital stays. Of note, the adoption of RASP has increased steadily in recent years, while the number of OSP procedures has decreased, and LSP procedures remain relatively stable. Moreover, based on our findings, a transcapsular approach to OSP seems to present slightly better peri‐operative outcomes in terms of length of hospital stay and intensive care unit admissions for patients with BPH and no concomitant bladder stones. Despite these advantages, each approach presents its own indications and contraindications, which must be carefully evaluated based on patient characteristics, surgeon expertise, and institutional resources.

Although robotic surgery is gaining traction in Germany, our data show that its overall use for simple prostatectomy remains limited, accounting for 3% of all cases in the last years. Nevertheless, this reflects a gradual increase in adoption, consistent with global trends observed in major urological procedures [18]. The growing interest in RASP can be attributed to its enhanced precision, reduced blood loss, and lower rates of peri‐operative complications, as previously reported [19]. However, the higher costs associated with robotic systems and the longer operating times remain important considerations that may limit their broader adoption, particularly in resource‐limited settings [20]. Additionally, the widespread availability and clinical effectiveness of laser enucleation techniques offer a well‐established alternative to RASP, further influencing its adoption in various healthcare environments [21]. In contrast, LSP has been adopted less extensively in Germany, even though it is associated with favourable peri‐operative outcomes [22]. Nevertheless, the available evidence suggests that RASP and LSP present similar peri‐operative and long‐term complication rates, as well as comparable functional outcomes and patient satisfaction rates [23].

Given that LSP and RASP are performed, in most cases, via a transcapsular approach, and considering that there is an OPS code only for transcapsular LSP and RASP, we compared LSP and RASP solely vs transcapsular OSP and not vs the transvesical approach [24, 25]. Major peri‐operative complications, such as transfusions, postoperative urinary retention, and length of hospital stay, were lower in patients undergoing minimally invasive simple prostatectomy compared to transcapsular OSP. Nevertheless, OSP remains a frequently performed procedure and is still preferred in specific clinical scenarios, such as for the management of significantly enlarged prostates or in centres with limited access to advanced surgical technologies [26]. Based on the previous notion, we also compared transcapsular vs transvesical OSP for patients with BPH without concomitant bladder stones. Our findings indicate that transcapsular OSP may present slightly better peri‐operative outcomes compared to transvesical OSP. It should be noted, however, that most OSPs are still performed via a transvesical approach in Germany [27].

It should also be highlighted that the median length of hospital stay after RASP or LSP is considerably shorter in many international series from other healthcare systems, often ranging between 1 and 2 days [28]. However, in Germany, prolonged lengths of hospital stay are influenced less by clinical necessity and more by structural and reimbursement‐related factors [29]. The German DRG system, under which hospitals are reimbursed, effectively incentivises longer inpatient stays [30]. Specifically, the reimbursement for urological surgeries such as RASP is tied to minimum length of stay thresholds. In other words, discharging patients significantly earlier than the average can result in reduced reimbursement [31]. Furthermore, cultural and systemic expectations play a role. German patients and providers often prioritise in‐hospital recovery and monitoring, particularly in older or multimorbid populations, which constitute a substantial proportion of BPH patients [32].

This study represents, to the best of our knowledge, the largest analysis comparing the peri‐operative outcomes and trends in OSP, LSP and RASP. However, several limitations must be acknowledged. First, the retrospective nature of the analysis, based on billing data, raises concerns about potential coding errors and misclassifications. Moreover, key clinical details, such as prostate volume, operating time, PSA values, Charlson Comorbidity Index, American Society of Anesthesiologists score, anticoagulation status, and further patient comorbidities, were not available and could not be assessed as confounders in the multivariable analysis. Data on long‐term outcomes, functional recovery, continence, and reoperation or readmission rates were also lacking. Moreover, it was beyond the scope of this study to assess peri‐operative mortality. Importantly, we did not aim to compare the peri‐operative outcomes of high‐volume centres performing simple prostatectomy. Lastly, our findings are specific to the German healthcare system, which may limit their generalisability to other countries with different healthcare infrastructures and surgical practices. Therefore, future research is warranted to explore the long‐term functional outcomes, cost‐effectiveness, and patient satisfaction associated with these surgical techniques.

Despite these limitations, these nationwide data highlight the growing adoption of RASP for the surgical management of BPH in Germany. Based on our analyses, RASP and LSP were associated with lower odds of transfusions, urinary retentions, and shorter hospital stays compared to OSP. However, these findings were derived from a retrospective, observational dataset with potential unmeasured confounders, therefore, they should be interpreted with caution. While the increasing adoption and improved peri‐operative outcomes suggest a shift towards minimally invasive surgery for BPH, OSP remains a valuable treatment modality that continues to play a significant role in specific clinical contexts. Every surgical approach has specific indications, advantages and limitations. These should be carefully discussed with patients to ensure tailored decision‐making and optimised outcomes.

## Author Contributions

All authors participated in the drafting, writing and editing of the manuscript.

## Ethics Statement

Written informed consent from the participants and ethical approval were not required for this study in accordance with the national legislation and institutional requirements. All data used in this work are stored anonymised at the German Federal Statistical Office.

## Disclosure of Interests

None declared.

## Funding Information

This research did not receive any specific grant from funding agencies in the public, commercial, or not‐for‐profit sectors.
